# Displacement of the Predominant Dengue Virus from Type 2 to Type 1 with a Subsequent Genotype Shift from IV to I in Surabaya, Indonesia 2008–2010

**DOI:** 10.1371/journal.pone.0027322

**Published:** 2011-11-07

**Authors:** Atsushi Yamanaka, Kris C. Mulyatno, Helen Susilowati, Eryk Hendrianto, Amor P. Ginting, Dian D. Sary, Fedik A. Rantam, Soegeng Soegijanto, Eiji Konishi

**Affiliations:** 1 Indonesia–Japan Collaborative Research Center for Emerging and Re-emerging Infectious Diseases, Institute of Tropical Disease, Airlangga University, Surabaya, Indonesia; 2 Center for Infectious Diseases, Kobe University Graduate School of Medicine, Kobe, Japan; 3 Department of International Health, Kobe University Graduate School of Health Sciences, Kobe, Japan; 4 BIKEN Endowed Department of Dengue Vaccine Development, Faculty of Tropical Medicine, Mahidol University, Bangkok, Thailand; Blood Systems Research Institute, United States of America

## Abstract

Indonesia has annually experienced approximately 100,000 reported cases of dengue fever (DF) and dengue hemorrhagic fever (DHF) in recent years. However, epidemiological surveys of dengue viruses (DENVs) have been limited in this country. In Surabaya, the second largest city, a single report indicated that dengue virus type 2 (DENV2) was the predominant circulating virus in 2003–2005. We conducted three surveys in Surabaya during: (i) April 2007, (ii) June 2008 to April 2009, and (iii) September 2009 to December 2010. A total of 231 isolates were obtained from dengue patients and examined by PCR typing. We found that the predominant DENV shifted from type 2 to type 1 between October and November 2008. Another survey using wild-caught mosquitoes in April 2009 confirmed that dengue type 1 virus (DENV1) was the predominant type in Surabaya. Phylogenetic analyses of the nucleotide sequences of the complete envelope gene of DENV1 indicated that all 22 selected isolates in the second survey belonged to genotype IV and all 17 selected isolates in the third survey belonged to genotype I, indicating a genotype shift between April and September 2009. Furthermore, in December 2010, isolates were grouped into a new clade of DENV1 genotype I, suggesting clade shift between September and December 2010. According to statistics reported by the Surabaya Health Office, the proportion of DHF cases among the total number of dengue cases increased about three times after the type shift in 2008. In addition, the subsequent genotype shift in 2009 was associated with the increased number of total dengue cases. This indicates the need for continuous surveillance of circulating viruses to predict the risk of DHF and DF.

## Introduction

Four types of dengue viruses (DENV1–4), mosquito-borne flaviviruses, are distributed throughout tropical and subtropical areas of the world, where approximately 2.5 billion people are at risk of infection. Infection with any of these types of virus causes dengue fever (DF) and its more severe form, dengue hemorrhagic fever (DHF), with an estimated 50–100 million cases and a reported 250,000–500,000 cases every year, respectively [1], [2]. Although infection with one type of DENV protects individuals from subsequent infection with the same type of DENV, secondary infection with a different type of DENV increases the risk of DHF [3].

DENVs of each type are grouped into several genotypes [4]. Phylogenetic studies have revealed that DENV1 comprise five genotypes: (I) Southeast Asia, China and East Africa; (II) Thailand; (III) sylvatic (Malaysia); (IV) West Pacific Islands and Australia; and (V) America, West Africa and Asia [5], [6]. These studies have demonstrated geographical movement of DENVs, divergence in particular areas and associations between particular genotypes and disease severity [7]–[10]. Displacements of DENV types, genotypes and clades have occurred in dengue-endemic countries [11]–[15], possibly initiated by imported cases [16], [17]. Moreover, previous reports demonstrated that displacements had been associated with changes in disease incidence and severity [14], [15], [18]. It is thus extremely important that molecular surveillance of circulating DENVs is carried out in dengue endemic countries to predict the impact of associated disease.

Indonesia has experienced approximately 100,000 annual cases of DF and DHF in recent years [19]. The first recorded dengue outbreak in Indonesia occurred in Java Island (Jakarta and Surabaya) in 1968 [20], [21]. Although all DENV types were isolated from patients in the Jakarta metropolis in 1973–1974 [22], subsequent molecular epidemiological surveys of circulating viruses in Indonesia have been limited. DENV3 has been the major endemic type of DENV in Jakarta during the past 20 years [19], [23]. In the second largest city in Indonesia, Surabaya (with a population of 3 million people residing in approximately 300 km^2^), only two epidemiological surveys of circulating DENVs have been performed and published. The first report indicated that 80% of villages in Surabaya were considered dengue-endemic areas in 1999 [24], but this study did not involve laboratory analyses such as virus isolation and typing. The first typing analysis was performed between 2003 and 2005 and revealed that DENV2 was predominant: of 25 patients, 20 (80%) were infected with DENV2, four (16%) with DENV3 and one (4%) with DENV4 [25]. (These data were contained in an unpublished thesis written in Indonesian; thus limiting accessibility.) However, no studies have been carried out on circulating DENVs in Surabaya over the last five years. Here, we report that the predominant DENV shifted from DENV2 to DENV1 in Surabaya between October and November 2008, followed by a genotype shift of DENV1 from IV to I between April and September 2009.

## Materials and Methods

### Serum samples

Serum samples were collected from 1071 patients aged from four months to 14 years, who were clinically diagnosed with DF or DHF at the Department of Child Health, Dr. Soetomo Hospital in Surabaya. All patients in this study were Surabaya inhabitants. Collections were made during: (i) April 2007, (ii) June 2008 to April 2009, and (iii) September 2009 to December 2010. Virus isolation was attempted on all samples. The infection history (primary or secondary infection) of these patients was determined based on the combined results of IgG and IgM antibody presence in acute sera using a Dengue Duo Cassette (Panbio, Sinnamon Park, Australia). Briefly, IgG or IgM antibodies in the patient sera bind to colloidal gold complexes containing recombinant DENV1–4 antigens, which provide visible reactions with IgG and/or IgM. Dr. Soetomo Hospital is a teaching hospital belonging to the Faculty of Medicine, Airlangga University, and the biggest provincial hospital in East Indonesia.

### Ethics statement

This study was approved by the Ethical Committees of Kobe University Graduate School of Medicine (Ethical Committee Approval Number 784) and Airlangga University (069/PANEC/LPPM/2009). Ethical approval obtained by Airlangga University included permission to use samples collected from Dr. Soetomo Hospital, since this hospital belonged to Airlangga University as a teaching hospital. Parents of all patients (aged from four months to 14 years) received a detailed explanation of the present study. Patients, or their parents, who agreed to the use of their blood samples and the publication of their cases, provided us with signed consent. Residual serum samples after the routine investigations were used. No specific permissions were required for mosquito collection in these locations, excepting that whenever mosquito collection was performed indoor we obtained permission from the owner of each private house.

### Mosquito samples

In April 2009, 750 mosquitoes (*Aedes aegypti*) were collected from a village (Simo Sidomulyo) in Surabaya. One hundred and fifty adults and 600 immature mosquitoes (larvae and pupae) were collected using sweeping nets and ladles, respectively. The immature mosquitoes were collected from 10 different breeding sites, separated from each other by >100 m. The breeding sites were mostly indoor water storage containers and outdoor discarded trash cans. Based on their gender and engorged conditions, the 150 adult mosquitoes were divided into six groups: three of engorged females, one of unengorged females and two of males (21–32 mosquitoes/group). The larvae were reared at 28°C±2°C in our laboratory, and then 1- to 3-day-old adults were divided into five groups for males and seven groups for females (50 mosquitoes/group). These 18 groups of mosquitoes were homogenized in 2 ml of cell culture medium (see below), and filtered through a 0.2 µm filter (Sartorius Biotech GmbH, Goettingen, Germany) prior to virus isolation.

### Virus isolation and typing

Vero cells used for virus isolation were cultured at 37°C in Eagle's minimum essential medium supplemented with 10% fetal bovine serum and 60 µg/ml kanamycin. Cells were inoculated with 10-fold diluted serum samples prepared in culture medium or filtered mosquito homogenates [26]–[28], and then incubated for 5–7 days. After five blind passages, cell monolayers were examined for the presence of viral antigen by immunostaining with a flavivirus-specific monoclonal antibody (D1–4G2; American Type Culture Collection, Manassas, VA), as described previously [26]. Viral RNA was extracted from the infected cells, and the type was determined by PCR using type-specific primers following a previous report [29].

### Phylogenetic analysis

The envelope (E) region, comprising 1485 nt, has been commonly used for phylogenetic analysis of dengue isolates because of its wide diversity [4], [5], [23]. The E nucleotide sequences were determined from DENV1 isolates using an ABI PRISM 310 Genetic Analyzer (Applied Biosystems, Foster City, CA). Phylogenetic analysis was performed using the neighbor-joining (NJ) method with a Kimura 2-parameter model (GENETYX version 9, Tokyo, Japan). Bootstrap analysis with 1,000 replicates was used to assess the reliability of the predicted trees. For homology searches using the Surabaya strains, the NCBI BLAST software version 2.2.23 (http://blast.ncbi.nlm.nih.gov/Blast.cgi) was used. The year of divergence into each clade was estimated using a relaxed molecular clock approach using the Bayesian Markov Chain Monte Carlo (MCMC) method available in the Bayesian Evolutionary Analysis by Sampling Trees (BEAST) software package v1.5.3 [30], incorporating information on virus sampling time. This analysis used a strict molecular clock, a General Time Reversible (GTR) + Γ_4_ model of nucleotide substitution for each codon position and a Bayesian skyline coalescent model (five coalescent-interval groups), the conditions for which were the same as previously reported for DENV analysis [31], [32]. Similar results, without major differences in topology or coalescent times, were obtained using a relaxed (uncorrelated lognormal) molecular clock and different substitution models (results available from the authors on request). All chains were run for a sufficient length of time, with 300 million generations and multiple time points to ensure convergence, with 10% removed as burn-in. This analysis allowed us to estimate times to the most recent common ancestor (TMRCA). The degree of uncertainty in each parameter estimate was provided by the 95% highest posterior density (HPD) values, while posterior probability values provided an assessment of the degree of support for each node on the tree.

### Statistical analysis

The association between DENV type and disease severity was evaluated by the chi-square test with the Yates' correction factor. The probability value of P⩽0.05 was considered statistically significant.

## Results

### Shift of the predominant DENV from type 2 to type 1

**Figure 1:**
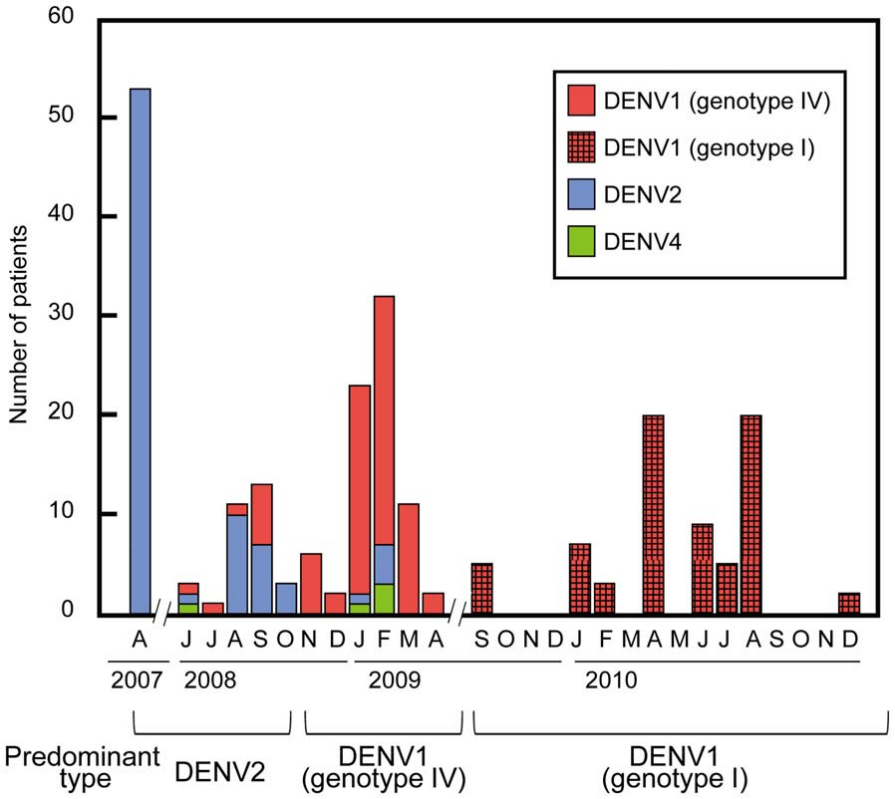
The monthly number of dengue isolates. The number of isolates is indicated monthly for each dengue virus (DENV) type between June 2008 and December 2010. Solid red bar, number of DENV1 genotype IV isolates; hatched red bar, number of DENV1 genotype I isolates; blue bar, DENV2 isolates; green bar, DENV4 isolates. In this study period, DENV3 was not isolated.

In the first and second surveys, immunostaining determined that a total of 160 isolates were infected with flavivirus. Fifty-three isolates collected in April 2007 (the first survey) were subjected to RT-PCR typing. DENV2 was exclusively detected (Fig. 1), consistent with a previous study reporting that DENV2 was predominant in 2003–2005. During June 2008 to April 2009 (the second survey), 107 isolates were obtained. In the first half (June–October 2008) of the survey, the profile of the isolated virus type was different from that obtained in our first survey (Fig. 1). Although DENV2 was still predominant (68%; 21 of the 31 patients), DENV1 was isolated in a considerable proportion of cases (29%; nine patients) and DENV4 was also isolated in one case (3%; one patient). In the second half of our survey (November 2008 to April 2009), DENV1 became the predominant type. Specifically, 67 of 76 patients (88%) were infected with DENV1, five patients (7%) were infected with DENV2, and four patients (5%) were infected with DENV4 (Fig. 1). This indicated that the predominant DENV shifted from DENV2 to DENV1 between October and November 2008 and that DENV1 had predominated since.

Of 18 pools of mosquito samples collected in April 2009, immunostaining showed that nine (three from adult and six from immature mosquitoes) were infected with flavivirus. The isolates were determined to be exclusively DENV1 by PCR typing. This indicated that a transmission cycle of DENV1 between humans and mosquitoes had already been established in Surabaya by April 2009.

### Phylogenetic analysis of DENV1 isolated in Surabaya

**Figure 2:**
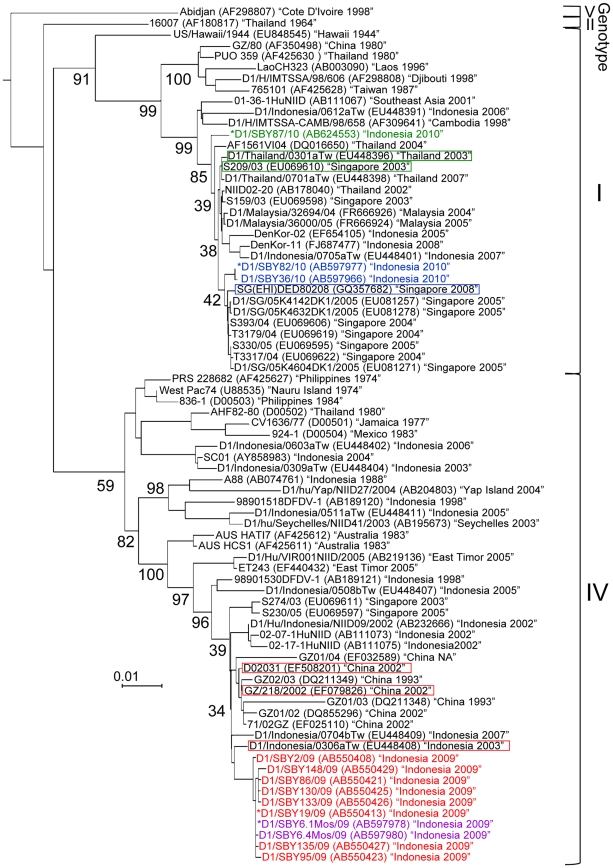
Phylogenetic tree based on the E gene sequence of DENV1 strains constructed using the neighbor-joining method. The GenBank accession number is described in parentheses, followed by the country and year in which the strains were isolated (in quotations). ‘NA’ indicates that the information was not available. The representative Surabaya isolates are indicated in red, purple, blue or green letters. For strains isolated from other areas available in the GenBank database, only selected strains which had been used in previous reports are shown [4], [5], [33]. Strains that showed the highest BLAST score against the Surabaya isolates are enclosed individually with red, blue or green squares. Major bootstrap values are shown at nodes, and the scale bar represents 0.01 nucleotide changes per site. Asterisks indicate isolates D1/SBY19/09, D1/SBY6.1Mos/09, D1/SBY82/10 and D1/SBY87/10, which represent the 15, 2, 11 and 2 Surabaya isolates, respectively, with an identical nucleotide sequence in the E region.

The nucleotide sequence of the E gene was determined in 22 randomly selected DENV1 isolates from the second survey (June 2008 to April 2009). This analysis indicated high conservation among these 22 isolates (99.6–100% similarity). The nucleotide sequences were identical among 15 isolates, as represented by isolate D1/SBY19/09. Thus, eight isolates (D1/SBY2/09, D1/SBY19/09, D1/SBY86/09, D1/SBY95/09, D1/SBY130/09 D1/SBY133/09, D1/SBY135/09 and D1/SBY148/09) were used for phylogenetic analyses and are indicated in red in Fig. 2. All of these isolates belonged to genotype IV (the West Pacific Islands and Australia group), according to the generally accepted classification system [5]. For intra-genotype comparisons, 20 strains were selected from the GenBank database (on June 5, 2011) that were phylogenetically close to the Surabaya isolates with high scores (2455–2671), as identified using the NCBI BLAST software. This phylogenetic tree also included all Indonesian genotype IV strains and representative genotype IV strains isolated outside Indonesia during the 1970s to 2000s that have commonly been used in previous reports [4], [5], [33].

**Figure 3:**
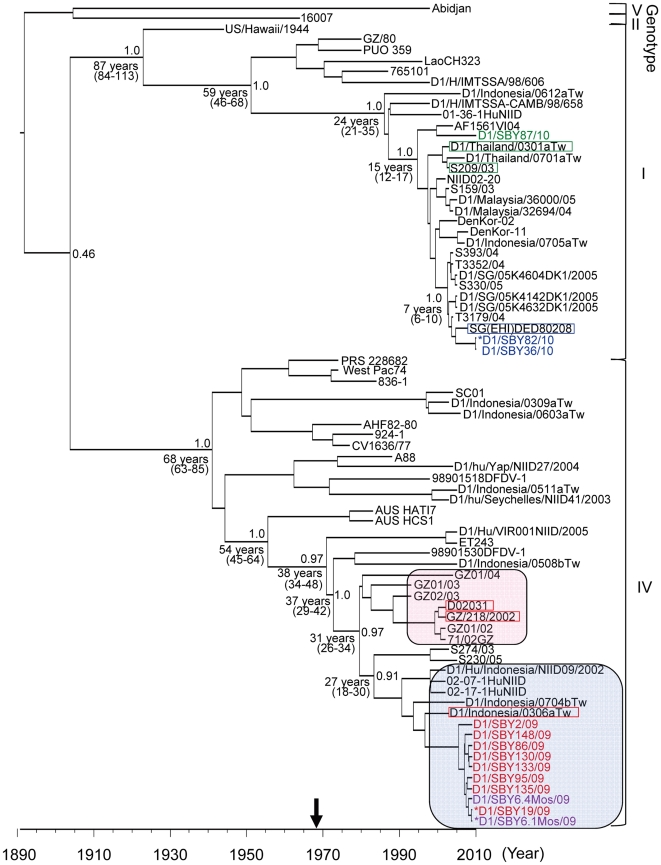
Maximum clade credibility (MCC) tree of the E coding region of DENV1. The representative Surabaya isolates are indicated in red, purple, blue or green letters, and strains with the highest BLAST score are enclosed individually with red, blue or green squares. Horizontal branches are drawn to a scale of the estimated year of divergence with tip times reflecting sampling date (year). The arrow on the horizontal line indicates the year of the first outbreak in Indonesia. The coalescent times of some key nodes, as well as their 95% HPD values, are shown. Posterior probability values of ≥0.9 are shown above nodes. The tree is automatically rooted under the assumption of a molecular clock. The Surabaya isolates are indicated in boldface and underlined. Strains that showed the highest BLAST score against the Surabaya isolates are underlined. Squares with blue and red backgrounds indicate the preliminarily designated Indonesia and China groups, respectively.

The Surabaya DENV1 isolates showed 93.1–99.1% similarity to the other genotype IV strains listed in the tree (Fig. 2). Three strains (D1/Indonesia/0306aTw, D02031 and GZ/218/2002: enclosed by a red square in Fig. 2) showed the highest BLAST score (2671) and were grouped into a clade with a bootstrap value of 34%, along with the Surabaya isolates. One strain, D1/Indonesia/0306aTw, was isolated in Taiwan as a case imported from Indonesia in 2003 [16], while the other two strains, D02031 [17], [34] and GZ/218/2002 (GenBank accession number: EU448408), were isolated in 2002 from Guangzhou city in Guangdong province, the most severely dengue-affected area in China [17], [35], [36]. The substitutions in the Surabaya DENV1 genotype IV isolates included 25 nucleotide changes in the E region compared with D1/Indonesia/0306aTw. Fifteen of these were translationally silent mutations. The most common nucleotide change found in the Surabaya isolates, was G to A at position 607, which changed Glu to Lys at position 203. The results of molecular clock analysis are shown in Fig. 3. The D1/Indonesia/0306aTw strain was included in a clade containing the Surabaya genotype IV strains (preliminarily designated the Indonesia group; shown in a square with a blue background in Fig. 3). Strains D02031 and GZ/218/2002 were included in a different clade containing other Chinese strains (preliminarily designated the China group; shown in a square with a pink background in Fig. 3). They shared the most recent common ancestor approximately 31 years ago (95% HPD of 26–34 years) with high posterior probability values (0.97).

Three strains were randomly selected from those isolated from mosquitoes in April 2009. The nucleotide sequence of the E gene was determined for these isolates and the information was added into the phylogenetic tree (Fig. 2). The nucleotide sequences in two of the three isolates were identical, with strain D1/SBY6.1Mos/09 being representative. As expected, D1/SBY6.1Mos/09 and the third isolate D1/SBY6.4Mos/09 (shown in purple in Fig. 2) were also classified into the clade of genotype IV, along with the isolates from patients. Isolate D1/SBY6.1Mos/09 shared an identical sequence with isolate D1/SBY19/09, which was representative of the 15 isolates from patients.

### Shift of the predominant DENV1 genotype from IV to I

During the third survey (September 2009 to December 2010), immunostaining determined that 71 samples were infected with flavivirus. DENV1 was exclusively detected from the 71 samples, which indicated that DENV1 had been maintained as the predominant viral type in Surabaya. Fifteen randomly selected strains isolated between September 2009 and August 2010 were also analyzed phylogenetically (Fig. 2). Of these 15 isolates, 14 showed an identical nucleotide sequence, and D1/SBY82/10 was used as a representative strain. D1/SBY82/10 and another strain D1/SBY36/10 (shown in blue in Fig. 2) were classified into genotype I (Southeast Asia, China and East Africa group) and were most closely related to the Singaporean strains isolated during 2004–2008 (99.3–99.6% similarity). D1/SBY82/10 showed the highest BLAST score (2896) with the Singaporean strain SG(EHI)DED80208 (enclosed by a blue square in Fig. 2) isolated in 2008 [37]. Comparison of the amino acid sequences between Surabaya DENV1 genotype I strains and SG(EHI)DED80208 showed that Ala at position 481 in the Singaporean strains changed to Val in isolates D1/SBY82/10 (C to T at position 1442) and D1/SBY36/10. As well as this substitution, Gly at position 17 in the Singaporean strains changed to Arg in isolate D1/SBY36/10. The genotype I Surabaya DENV1 isolates showed 94.2–99.6% similarity to the other genotype I strains listed in the tree (Fig. 2). The most recent common ancestor from the Indonesian and Singaporean strains might have existed approximately seven years ago (95% HPD of 6–10 years; Fig. 3).

Two strains isolated in December 2010 showing identical nucleotide sequences (for which D1/SBY87/10 was representative, shown in green in Fig. 2) also belonged to genotype I (Fig. 2). However, these strains were not classified into the same clade as the D1/SBY82/10 strain isolated before August 2010. The degree of similarity to isolates D1/SBY82/10 and D1/SBY36/10 was 98.5–98.6%. Two amino acids in D1/SBY87/10 were substituted in isolates D1/SBY82/10 and D1/SBY36/10: changing Val to Ile at position 320 (G to A at position 958) and Val to Ala at position 481 (T to C at position 1442). As shown in Fig. 3, the common ancestor might have diverged approximately 15 years ago (95% HPD of 12–17 years). D1/SBY82/10 showed the highest BLAST score (2687) with the Thai and Singaporean strains (D1/Thailand/0301aTw and S209/03: enclosed by a green square in Fig. 2) isolated in 2003 [16].

### Clinical investigation

A total of 107 patients recruited in our second survey (from June 2008 to April 2009), for which DENV infection had been confirmed by PCR typing, were analyzed with regard to their clinical outcomes. As shown in Table 1, 43 (57%) of the 76 patients infected with DENV1 displayed DHF, which conferred a significantly higher risk of severe manifestation than those of DENV2 (15%, 4 of 26; P<0.005), but not DENV4 (40%, 2 of 5; P>0.05). Furthermore, 34 (79%) of the 43 DHF patients with DENV1 were secondary infections, which were significantly more severe than primary infections with DENV1 (21%, 9 of 43; P<0.025). On the other hand, the nine DHF patients (21%) with primary DENV1 infection also implied that the DENV1 genotype IV strain carried a higher virulence rate and indeed these patients showed a significantly higher risk for increased disease severity than those with DENV2 primary infection (P<0.025).

**Table 1:** The number of patients infected with each dengue virus (DENV) type and their clinical diagnosis, during 2008–2009.

Diagnosis^1^	History^2^	# of patients		
		DENV1	DENV2	DENV4
**DF**	Primary	15	12	1
	Secondary	18	10	2
Subtotal		33	22	3
**DHF**	Primary	9	0	0
	Secondary	34	4	2
Subtotal		43	4	2
** Total**		76	26	5

1 A total of 107 patients were diagnosed with dengue fever (DF) or dengue hemorrhagic fever (DHF).

2 The dengue infection history was determined as primary or secondary infection by comparison between IgG and IgM antibody levels.

## Discussion

This study shows a quick type shift of the predominant circulating DENV from DENV2 to DENV1 in Surabaya between October and November 2008 (Fig. 1). Statistics by the Surabaya Health Office indicated that 10% of dengue cases were linked with DHF in 2008, increasing to 28% in 2009. The introduction of DENV1 into the DENV2-endemic area of Surabaya may have increased the opportunity for secondary heterotypic infections. The statistical results of our clinical investigations, indicating that patients with DENV1 secondary infection have a significantly higher risk for disease severity than those with DENV1 or DENV2 primary infections, would appear to support the statistics held by the Surabaya Health Office. We also found a DENV1 genotype shift from IV to I in Surabaya between April 2009 and September 2009, less than one year after displacement of the viral type. According to statistics by the Surabaya Health Office, the proportion of DHF in the total dengue cases in 2010 was approximately 13%, which was decreased compared with 2009 (28%). However, the total number of dengue patients increased in 2010, with 2169, 2268 and 3379 cases in 2008, 2009 and 2010, respectively. This indicated that the genotype shift might be one of the factors contributing to the increased number of dengue cases between 2009 and 2010 in Surabaya.

The association of the DENV type, and infection status (primary or secondary), with disease severity is variable and unique to each country and strain [38]–[42]. But, frequently displacements of DENV type, genotype or clade can be related to an increased risk of severe disease [14], [15], [18]. Although the mechanism of the displacement is still unclear, selection by host immunity and viral evolution may be involved [43], [44]. As observed in our study, previous reports have also shown the relationship between DENV1 and increased severity [41], [42]. Other reports indicate that a clade replacement within the same genotype was associated with the emergence of DHF [14]. The recent Surabaya strain (D1/SBY87/20), isolated from patients in December 2010, was allocated to a different clade from that of the previous isolates (D1/SBY82/10 and D1/SBY36/10) (Figs. 2 and 3), suggesting that a clade shift may have occurred in 2010. Although we did not see an emergence of DHF in 2010, there is a need for careful monitoring of the changes in DENV type in order to be prepared for future increase in the incidence of DHF in Surabaya.

Although this study has highlighted a rapid displacement in one DENV endemic area (Surabaya), it is reasonable to suppose that other endemic areas in Indonesia are also constantly exposed to the risk of viral displacement, as reported in several other countries [11]–[16]. DENV2 strains isolated in Surabaya in 2008 were phylogenetically closely related to those isolated from Sumatra Island in Indonesia (GenBank accession numbers: AB189122, AB189123 and AB189124; data not shown). Similar DENV2 Sumatra strains were isolated in 1998, during a dengue outbreak in Indonesia [45], suggesting that this lineage had been distributed widely for some time in Indonesia.

Surabaya DENV1 isolates from November 2008 to April 2009 (shown in red in Figs. 2 and 3) were grouped into the genotype IV clade which included: five Indonesian strains (shown in a square with a blue background in Fig. 3); 02–17–1HuNIID, 02–07–1HuNIID and D1/Hu/Indonesia/NIID09/2002 isolated from areas other than Surabaya; and D1/Indonesia/0306aTw and D1/Indonesia/0704bTw isolated as imported cases from Indonesia [34], [46]. However, genome sequence analysis revealed that the Surabaya isolates were most closely related to the Chinese strain (D02031) isolated from Guangzhou city in Guangdong province in 2002 (enclosed by a red square in Figs. 2 and 3). The low bootstrap value (34%) between the Chinese and Indonesian strains indicated their close relationship, suggesting the introduction into Surabaya of a strain that had circulated in China or other areas of Indonesia. BEAST analysis, for estimating the year of virus divergence, enabled us to speculate about virus emergence and spread. The most recent ancestor (27 years ago) of the Surabaya genotype IV strains may have existed two decades earlier than that of the Surabaya genotype I strains, D1/SBY82/10 and D1/SBY36/10 (7 years ago; Fig. 3). Approximately 40 years ago (in 1968; indicated by the arrow in Fig. 3), the first dengue outbreak occurred in Indonesia and the virus quickly spread throughout this archipelago [20]–[22]. The ancestors of the genotype IV virus seemed to frequently diverge after the outbreak, with each strain then potentially evolving individually in each area of Indonesia. Although the Surabaya isolates were found to be phylogenetically linked to the Chinese and Indonesian strains by the NJ method (Fig. 2), the Bayesian MCMC method showed that the Surabaya isolates formed a clade (designated the Indonesian clade), along with another five Indonesian strains (shown in a square with a blue background in Fig. 3). These results indicated that the DENV1 strain that displaced DENV2 in Surabaya in 2008 might have been transported from another dengue endemic area in Indonesia.

Our findings also revealed that Surabaya genotype I strains isolated in 2010 (except for D1/SBY87/10) showed high nucleotide similarity to the Singaporean strains. In contrast to the situation in Surabaya, a displacement from DENV1 to DENV2 occurred in Singapore between 2006 and 2007 [37]. DENV1, the previous predominant type in Singapore, caused an outbreak in 2005. The DENV1 Singaporean strain SG(EHI)DED80208, to which the present Surabaya isolates were most closely related, was similar to the strains responsible for the 2005 outbreak in Singapore [37]. This suggested the possibility of a DENV1 outbreak in Surabaya, similar to that observed in Singapore. The ancestor of the genotype I viruses (with the exception of D1/SBY87/10) existed up until 7 years ago, and the neighbors of the Surabaya genotype I strains were all Singaporean strains, as demonstrated by our phylogenetic trees (Fig. 2 and 3). These data suggest that the genotype I strain that displaced the genotype IV strains in Surabaya in 2009 might have been transported from Singapore. It is likely that D1/SBY87/10 was an introduced strain and did not emerge by sequence divergence from local strains in Surabaya, since the substitution of more than 20 nucleotides in the E coding region is unlikely to have occurred in 4–5 months [47]. As for the recent Surabaya strain (D1/SBY87/10), its ancestor obviously existed 15 years ago, making it older than D1/SBY82/10 and D1/SBY36/10, suggesting that D1/SBY87/10 was also introduced from another area in Indonesia or from another country altogether.

In conclusion, this is the first report describing the molecular epidemiology of DENVs circulating in Surabaya. Our results indicated that DENV1 displaced DENV2 in Surabaya in 2008 with a subsequent genotype shift from IV to I. The shift of DENV types and genotypes may have increased, and will potentially continue to increase, the proportion of DHF cases among the total number of dengue cases, as well as the total number of dengue patients. Thus, Surabaya inhabitants need to be vigilant against mosquitoes to avoid the risk of contracting DHF.
